# Downregulation of microRNA-100 protects apoptosis and promotes neuronal growth in retinal ganglion cells

**DOI:** 10.1186/s12867-014-0025-1

**Published:** 2014-11-18

**Authors:** Ning Kong, Xiaohe Lu, Bin Li

**Affiliations:** Department of Ophthalmology, Zhujiang Hospital, Southern Medical University, Guangzhou, 510280 Guangdong Province China; Department of Ophthalmology, Guangzhou Panyu Central Hospital, Guangzhou, 510280 Guangdong Province China

**Keywords:** Retinal ganglion, miR-100, Oxidative stress, Apoptosis, IGF-1

## Abstract

**Background:**

Retinal ganglion cells (RGCs) are preferentially lost in glaucoma or optic neuritis. In the present study, we investigated the protective effect of mircoRNA 100 (miR-100) against oxidative stress induced apoptosis in RGC-5 cells.

**Results:**

Rat RGC-5 cells were cultured in plates and H_2_O_2_ was added to induce oxidative stress. TUNEL assay and qRT-PCR showed H_2_O_2_ induced apoptosis and up-regulated miR-100 in a dose-dependent manner in RGC-5 cells. Conversely, lentiviral-mediated miR-100 down-regulation protected H_2_O_2_ induced apoptosis, promoted neurite growth and activated AKT/ERK and TrkB pathways through phosphorylation. Luciferase assay confirmed that IGF1R was directly regulated by miR-100 in RGC-5 cells, and siRNA-mediated IGF1R knockdown activated AKT protein through phosphorylation, down-regulated miR-100, therefore exerted a protective effect on RGC-5 apoptosis.

**Conclusion:**

Down-regulating miR-100 is an effective method to protect H_2_O_2_ induced apoptosis in RGC-5 cells, possible associated with IGF1R regulation.

## Background

Retinal ganglion cells (RGCs) are a group of specialized sensory neurons in the central nervous system, rested in the inner layer of the retina. RGCs capture visual signals from bipolar and amacrine cells through the photoreceptors on the outer retina, and electrically transmit that information to the brain. In glaucoma patients, increased intraocular pressure damage the cell bodies as well as the axons of RGCs, resulting in permanent and irreversible blindness [1]. The degeneration of retina in glaucoma patients is often accompanied by apoptosis of RGCs, which may be caused by excitotoxicity, hypoxia, or oxidative stress [2-4]. Many of the molecular pathways, including Akt, ERK, insulin-like growth factor 1 (IGF-1) and its receptor IGF1R, brain-derived neurotrophic factor (BDNF) and its tyrosine kinase receptor TrkB, repulsive guidance molecule, RGMa and its receptor neogenin, are actively involved in the development or the protection/regeneration of sensory neurons including RGC [5-13]. However, though many of the molecular pathways have been identified to protect RGCs from degeneration and induce optical nerve regeneration [14], the exact mechanism of RGC damage, or feasible clinical strategy for RGC regeneration are largely unknown.

MicroRNAs (miRNAs) are a group of noncoding RNAs with ~20 nucleotides, that inhibit endogenous gene expression through translational cleavage [15]. In both animal and human visual systems, miRNAs have been demonstrated to be involved in various aspects of retina development, including retinogenesis, retinal homeostasis, or retinal damage. For example, studies in mutant mice have shown that, miR-183/96/182 clusters and miR132/212 were essential for the synaptic development in retina [16-18], and miR-124 was critical for the maturation of cone cells [19] in retina. Study also showed that miR-218 inhibits Robo/Slit pathway to maintain normal vascular function in retina [20]. In retinal ganglion cells, a recent study specifically demonstrated that miR-132 was involved in BDNF-mediated retinal ganglion axonal branching and maturation [21]. Specifically, emerging evidences demonstrate that miRNAs are highly associated with many aspects of retinal degeneration [22,23], yet the exact mechanisms of miRNAs on regulating retinal apoptosis or degeneration are largely unknown. MicroRNA-100 (miR-100), a member of miR-99 family (including miR-99a, miR-99b, miR-100), is a key apoptotic regulator in various cell types [24,25]. In retina, miR-100 was found to be associated with diabetic retinopathy, a common retinal disease leading to total blindness [26]. However, the function role of miR-100 in mediating retinal development or pathology remains elusive.

In the present study, we used an *in vitro* culture system to induce oxidative stress by H_2_O_2_ application in rat ganglion cell line RGC-5 cells. We found that microRNA 100 (miR-100) was specifically upregulated in RGC-5 cells upon apoptosis. We then investigated the anti-apoptosis effect of down-regulating miR-100, as well as the association between miR-100 down-regulation and apoptosis-related pathways, Akt/ERK/TrkB and IGF1R, in retina ganglion cells.

## Methods

### Ethics statement

All experimental procedures are approved by the Ethics Committee at Guangzhou Panyu Central Hospital, Guangzhou, China.

### RGC-5 cell culture

The RGC-5 cell line, a transformed retinal ganglion progenitor cell line originally generated by Dr. N. Agarwal at the University of North Texas Health Science in the United States, was obtained from the Zhonghan Ophthalmic Center in Guangzhou, China. RGC-5 cells were cultured in low-glucose Dulbecco’s modified Eagle’s medium (DMEM, Gibco, USA) containing 10% fetal calf serum (FBS, Sigma Aldrich, USA), 100 U/ml penicillin and 100 μg/ml streptomycin in a 37°C tissue culture chamber supplied with 95% air and 5% CO_2_. The RGC-5 cells were passaged by trypsinization every 2 to 3 days.

### Apoptosis induction

To induce apoptosis in RGC-5 cell, an oxidative stress model was used. Briefly, RGC-5 cells were seeded into 24-well plates at concentration of 1 × 10^5^/cm^2^. Various concentrations of hydrogen peroxide (H_2_O_2_) (100 μm ~1000 μm) were added into the culture for 24 hours.

### TUNEL assay

RGC-5 cells were fixed with 4% paraformaldehyde (PFA) in phosphate-buffered saline (PBS) for 20 min, and permeabilized with 1% Triton X-100 for 10 min. TUNEL staining was conducted with an In-Situ Apoptosis Detection Kit (Chemicon, USA) according to the manufacturer’s protocol. A primary antibody against neural progenitor marker Brn3a (1:500, Santa Cruz, USA) was also used to identify RGC-5 cells. Imaging was done on an Axiovert 200 fluorescence microscope (Carl Zeiss, Germany). For each condition, 5 ~ 8 regions (~100 cells per region) were randomly taken for analysis. The percentage of TUNEL-positive RGC-5 cells were calculated against all brn3a positive cells and normalized to the control condition. Each experiment was repeated at least 3 times.

### RNA isolation and quantitative real-time reverse transcription-PCR (qRT-PCR)

Cultured RGC-5 cells were trypsinized and collected. RNA and miRNA fractions were then extracted using a Trizol RNA purification Kit according to manufacturer’s instruction (Qiagen, USA). Total RNA concentrations were confirmed with a NanoDrop ND-1000 spectrophotometer (NanoDrop Technologies, USA) at 260 and 280 nm (A260/280), and analyzed with an Agilent 2100 Bioanalyzer (Agilent Technologies, USA). Quantitative real-time reverse transcription-PCR (qRT-PCR) was performed by a TaqMan miRNA Assay according to manufacturer’s instruction (Applied Biosystems, USA). The amplification conditions were 35 cycles of 20 s at 97°C and 2 min at 56°C. The internal housing keeping genes were U6 for miR-100, and GAPDH for insulin-like growth factor 1 receptor (IGF1R) respectively.

### Lentivirus transfection

The oligonucleotides of rno-miR-100 inhibitor, rno-miR-100 mimics and non-specific control were puchased from Ribo-Bio (Ribo-Bio, Shanghai, China). The coding sequences were then amplified and cloned into pCDH-CMV-MCS-EF1-coGFP constructs (System Biosciences, USA) to construct miR-100 inhibitor vector (miR100-Inhibitor), miR-100 mimics vector (miR100-mimics) and non-specific miRNA vector (miR-NC). The lentiviral vectors were co-transfected with pPACK packaging plasmid into 293 T cells. The viral products were then collected and titered. The transfection of miR-100-Inhibitor or miR-NC into RGC-5 cells was conducted by a Lipofectamine 2000 reagent according to manufacturer’s recommendation (Invitrogen, USA). The culture medium was changed 24 hours after transfection.

### Immunocytochemistry

RGC-5 cells were fixed with 10% neutral-buffered formalin containing 5% methanol for 10 minutes, followed by washing in PBS (3 × 10 mins). The cells were then treated with permeabilization solution containing 0.3% trition X-100 and 3% horse serum in PBS for 30 minutes, followed by washing PBS (3 × 10 mins). The cells were incubated with primary antibody of Thy-1 (1:250 in permeabilization solution, Sigma Aldrich, USA) at 4°C overnight in a humidified box. On second day, cells were incubated with secondary antibody of Alexa-Fluor 488 (1:500 in PBS) for 12 hours. RGC-5 cells were also stained with the fluorescent nuclear binding label 4’,6-diamidino-2-phenylindole (DAPI; 500 ng/mL).

### Western blot analysis

RGC-5 lysates of were prepared with a lysis buffer containing 50 mM Tris (pH 7.6), 150 mM NaCl, 1 mM EDTA, 10% glycerol, and 0.5% NP-40 and protease inhibitor cocktail (Invitrogen, USA). The total protein were then separated on 10% SDS-PAGE gel and transferred to the nitrocellulose membranes. We used primary antibodies against, Thy-1 (1:2000, Santa Cruz Biotechnology, USA), ERK1/2 (1:1000, Cell Signaling Technology, USA), phospho-Erk1/2 (pERK1/2, 1:2000, Cell Signaling Technology, USA), PI3k3 /serine-threonine kinase (AKT, 1:1000, Cell Signaling Technology, USA), phosphor-AKT (pAKT, 1:1000, Cell Signaling Technology, USA), TrkB (1:1000, Cell Signaling Technology, USA), phosphorylated TrkB (1:200, Cell Signaling Technology, USA) and IGF1R (1:200, Santa Cruz Biotechnology, USA). Horseradish peroxidase-conjugated secondary antibodies (Bio-Rad, USA) were used and Actin was used as internal control. The western blots were visualized with an enhanced chemiluminesence system (Amersham Biosciences, USA) according to the manufacturer’s protocol. Band intensities normalized to the total protein immuno-precipitation under each control condition and quantified with an ImageJ software (NIH, USA).

### Luciferase reporter assay

**Figure 1:**
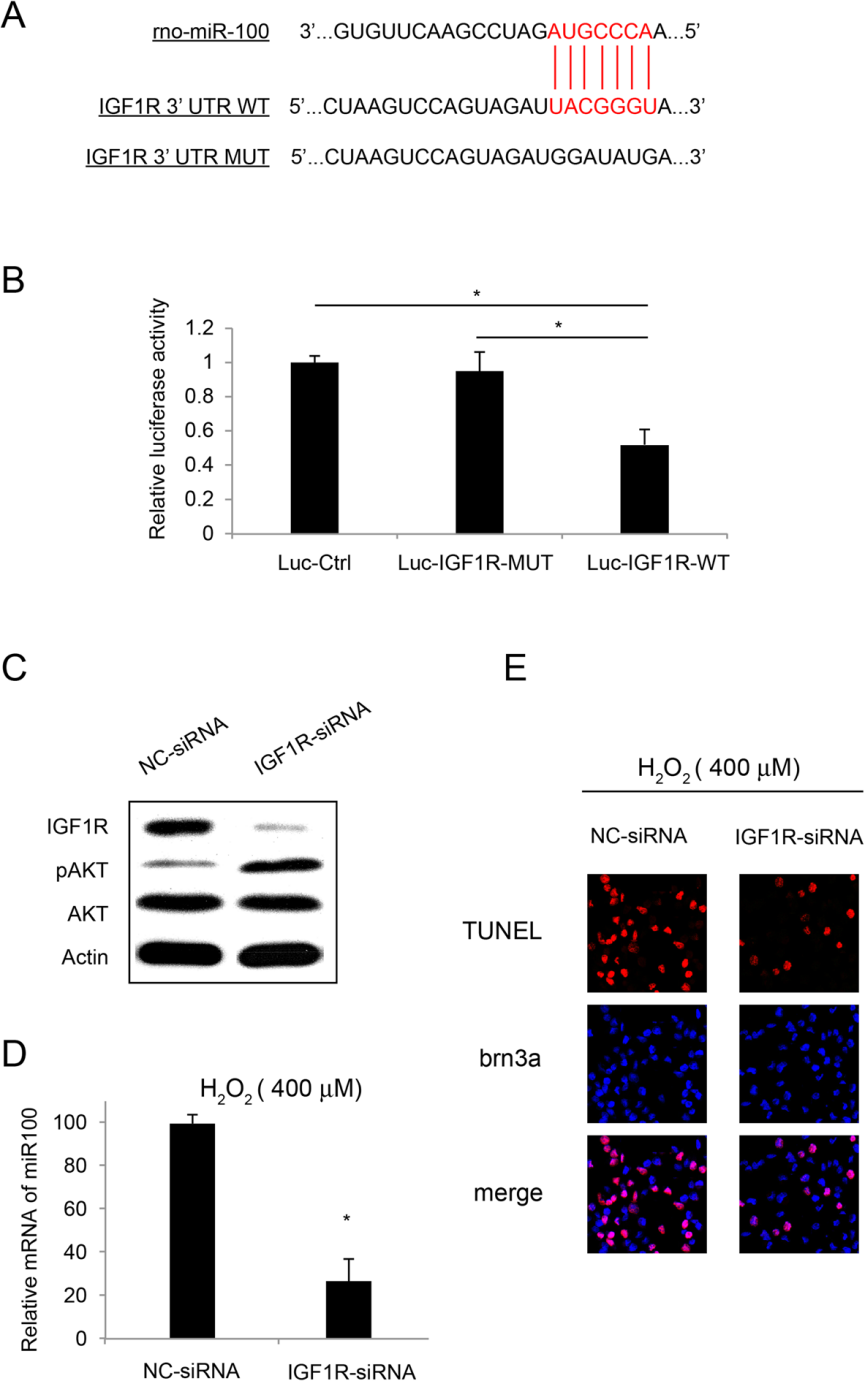
**miR-100 interacted with IGF1R in RGC-5 cells. (A)** Schematic diagram was shown for the predicted binding between rno-miR-100 and IGF1R 3’-UTR. The mutated 3’-UTR of IGF1R (IGF1R-mu) was also demonstrated. **(B)** In a luciferase report assay, HEK 293 T cells were transfected with pmiR-REPORT control vector (Luc-Ctrl), IGF1R vector with mutated 3’-UTR (Luc- IGF1R -mu) or IGF1R with wild-type 3’-UTR (Luc- IGF1R), along with β-galactosidase and miR-100 mimics for 24 hours. Luciferase signals were measured and normalized to the signal of control vector. (*: *P* <0.05). **(C)** RGC-5 cells were treated with IGF1R-siRNA or its non-specific siRNA (NC-siRNA), followed by western blotting on AKT/pAKT and IGF1R in 48 hours. RGC-5 cells were pre-treated with IGF1R-siRNA (100 nM) or its non-specific siRNA (NC-siRNA, 100 nM) for 24 hours, followed by H_2_O_2_ (400 μM) treatment for another 24 hours. **(D)** The mRNA expression level of miR-100 was assessed by qRT-PCR (*: *P* <0.05). **(E)** RGC-5 apoptosis was assessed by TUNEL staining.

RGC-5 cells were collected and regular PCR was performed to amplify the cDNAs of wild-type 3’-UTR of IGF1R. A mutant (MT) 3’-UTR of IGF1R (with modified binding site to rno-miR-100, Figure 1A) was also created by Site-Directed Mutagenesis Kit (SBS Genetech, China). The amplified cDNAs were then inserted into a pmiR-REPORT luciferase reporter vector (Ambion, USA) to construct Luc- IGF1R and Luc- IGF1R -mu vectors. The sequences of the vectors were verified by DNA sequencing. A non-specific control luciferase vector, Luc-Ctrl was also constructed. All three vectors were co-transfected with β-galactosidase and miR-100 mimics (Ribo-Bio, Shanghai, China) into HEK293 cells in 6-well plates by using Lipofectamine 2000 according to the manufacturer’s protocol. Twenty-four hours after transfection, the activity was assessed by a luciferase reporter assay system (Promega, USA) according to the manufacturer’s recommendtaion. All luciferase activities were normalized to the β-galactosidase signal under luc-Ctrl condition.

### siRNA transfection

The gene silencing IGF1R siRNA (IGF1R-siRNA) and non-specific control siRNA (NC-siRNA) were purchased from Stanta Cruz (Santa Cruz Biotechnology, USA). The transfection of siRNAs in RGC-5 cells was conducted with a Lipofectamine 2000 reagent according to manufacturer’s recommendation. After reaching 50% ~60% confluence, RGC-5 cells were transfected with IGF1R-siRNA (100 nM) or NC-siRNA (100 nM). The effect of siRNA on knocking down IGF1R protein, as well as other related proteins were examined by western blotting 48 hours after transfection.

### Statistical analysis

All data were shown as the mean ± S.E.M. For statistical analysis, a windows-based SPSS software was used. Comparison was made with student’s *t*-test and the difference was termed to be significant if *P* <0.05. All experiments were conducted in triplicates.

## Results

### H_2_O_2_ induced apoptosis and upregulated miR-100 in RGC-5 cells in a dose-dependent manner

**Figure 2:**
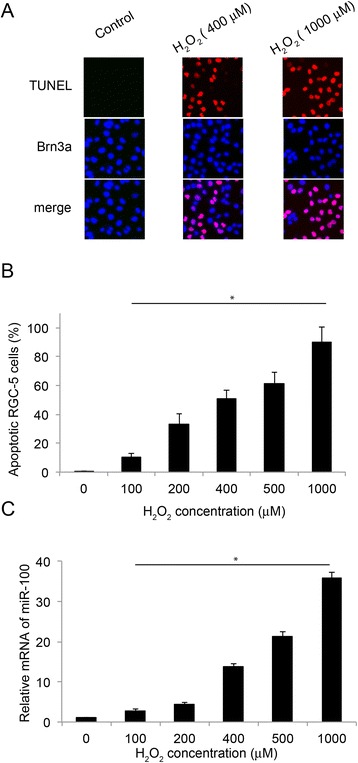
**H**_**2**_ **O**_**2**_ **induced apoptosis and upregulated miR-100 in RGC-5 cells.** RGC-5 cells were treated with various concentrations of H_2_O_2_ for 24 hours to induce apoptosis. **(A)** Representative fluorescent images were shown for the untreated RGC-5 cells (control), and RGC-5 cells treated with 400 μM or 1000 μM H_2_O_2_. An antibody against Brn3a (blue) was used to identify RGC-5 cells, and TUNEL staining was applied to identify apoptotic cells among them. **(B)** Quantification of the percentage of apoptotic RGC-5 cells (*: *P* <0.05, as compared to control). **(C)** The expression levels of miR-100, corresponding to the application of various concentrations H_2_O_2_, were measured by qRT-PCR (*: *P* <0.05, as compared to control).

First, we investigated the effect of oxidative stress on RGC-5 cell apoptosis. In our study, we cultured RGC-5 cells in 6-well plate for 24 hours, then included different concentration of H_2_O_2_ (100, 200, 400, 500 and 1000 μM) in RGC-5 cell culture for another 24 hours, followed by immunocytochemistry. The apoptosis was assessed by TUNEL assay. RGC-5 cells were identified by an antibody against Brn3a, a RGC-5 specific marker. Our results showed that H_2_O_2_ at the concentrations of 400 and 1000 μM induced significant apoptosis among RGC-5 cells (Figure 2A). Further analysis by quantifying the percentage of TUNEL-positive cells among all Brn3a-positive RGC-5 cells revealed that the apoptosis in RGC-5 cells was induced by H_2_O_2_ in a dose dependent manner (Figure 2B, *: *P* <0.05). We then looked at the effect of H_2_O_2_-induced apoptosis on the expression level of miR-100 in RGC-5 cells. Our results demonstrated that miR-100 was significantly up-regulated, and also in a dose-dependent manner by the application of H_2_O_2_ in RGC-5 cells (Figure 2C, *: *P* <0.05).

### Downregulation of miR-100 reduces H_2_O_2_ induced apoptosis and promoted neurite growth in RGC-5 cells

**Figure 3:**
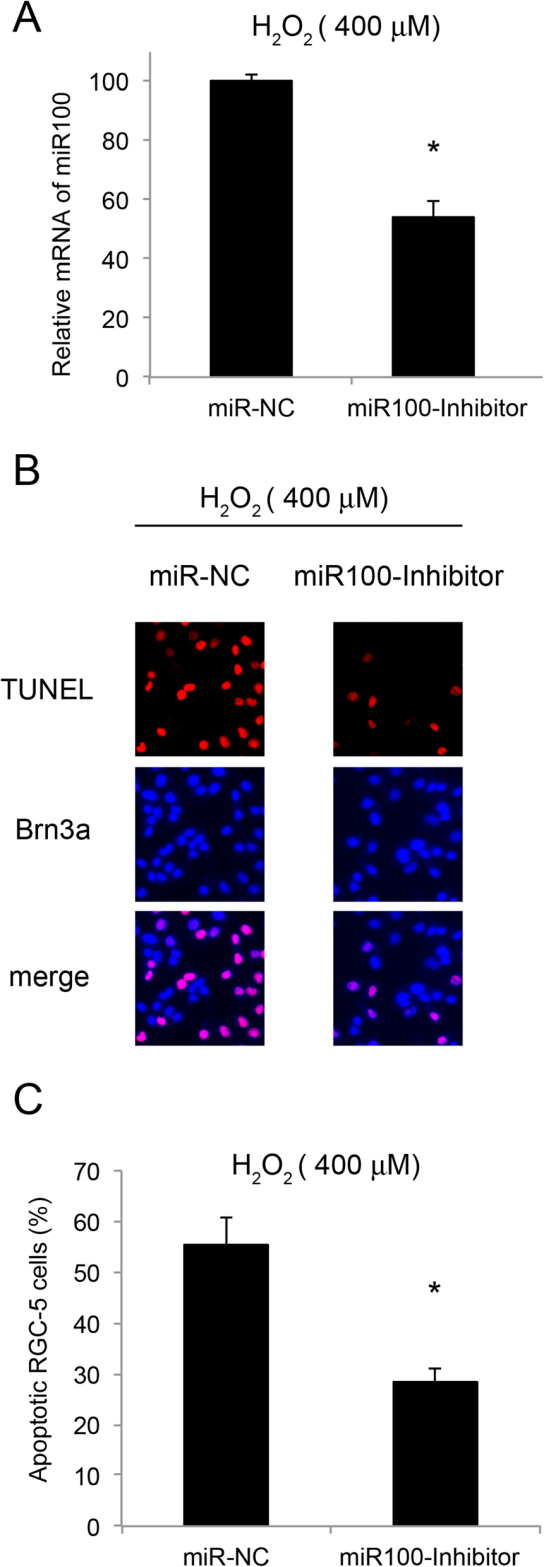
**Downregulation of miR-100 protected apoptosis in RGC-5 cells.** RGC-5 cells were pre-treated with lentiviral vector of miR100-inhibitor, or its non-specific miRNA vector (miR-NC) for 24 hours, followed by H_2_O_2_ (400 μM) treatment for another 24 hours. **(A)** The efficiency of down-regulating miR-100 by lentiviral vectors was evaluated by qRT-PCR (*: *P* <0.05). The effect of pre-treating RGC-5 cells with miR-100 down-regulation on oxidative stress induced apoptosis was then examined by TUNEL assay. **(B)** TUNEL staining was performed and representative fluorescent images were shown for the RGC-5 cells pre-treated with miR-NC, or miR100-inhibitor, and then H_2_O_2_ 400 (μM). **(C)** The percentages of apoptotic RGC-5 cells were also quantified (*: *P* <0.05).

We then investigated whether miR-100 played a functional role in modulating H_2_O_2_-induced apoptosis in RGC-5 cells. For that purpose, we constructed lentiviral vector expressing the inhibitory oligonucleotides of miR-100 (miR100-inhibitor) to specifically knock down the endogenous expression of miR-100. The efficiency of down-regulating miR-100 by lentivirus was verified by qRT-PCR. In that experiment, RGC-5 cells were transfected with either miR100-inhibitor, or its non-specific miRNA (miR-NC) for 24 hours. The results of qRT-PCR showed that the endogenous expression level of miR-100 was significantly and specifically down-regulated by miR100-inhibitor (Figure 3A, *: *P* <0.05).

To evaluate the effect of down-regulating miR-100 on RGC-5 apoptosis, we pre-treated RGC-5 cells with either miR100-inhibitor or miR-NC for 24 hours, followed by H_2_O_2_ (400 μM) treatment for another 24 hours. Our results of TUNEL staining showed that significantly less apoptosis were observed in miR-100 down-regulated RGC-5 cells than in control RGC-5 cells (Figure 3B). Quantification results demonstrated that the percentage of apoptotic cells reduced from 55.5% in control RGC-5 cells to around 30% in cells with miR-100 down-regulation (Figure 3C, *: *P* <0.05).

**Figure 4:**
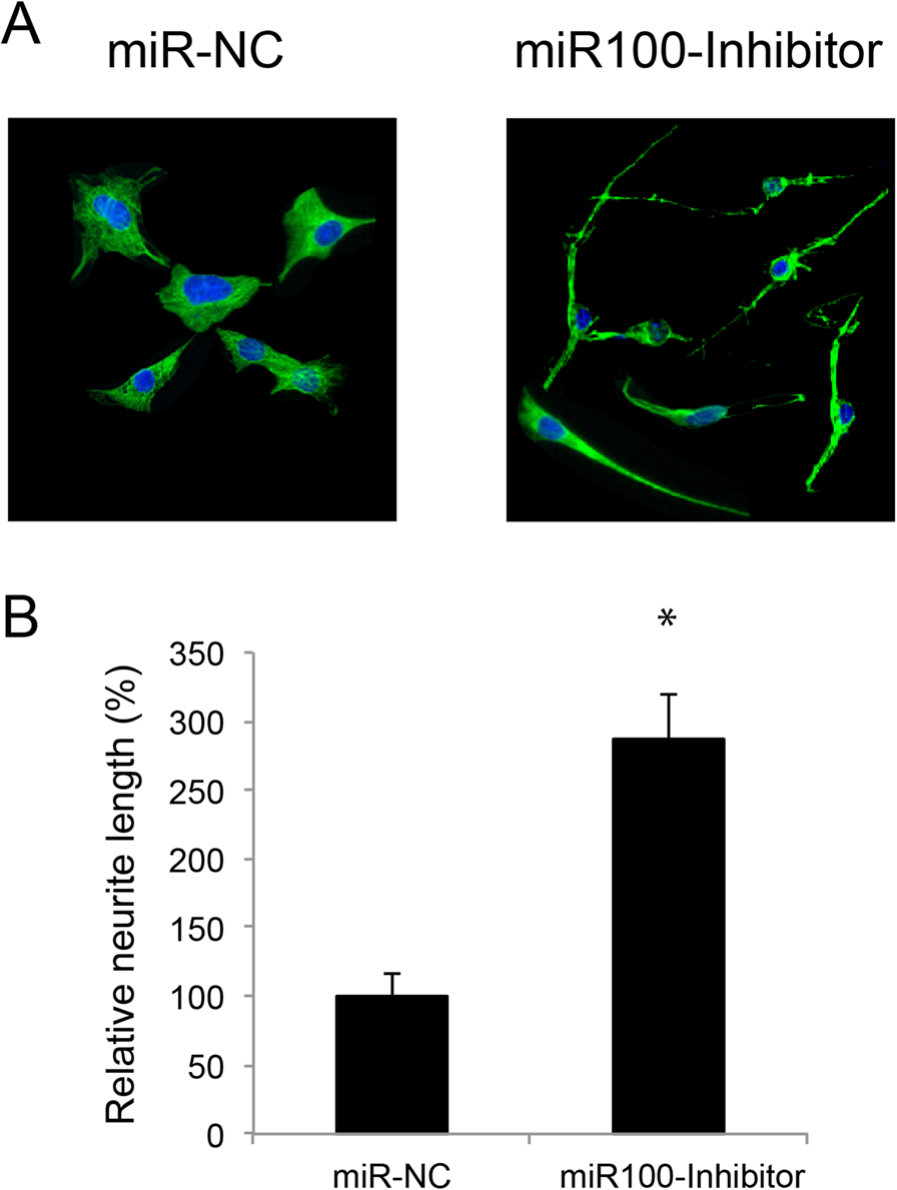
**Downregulation of miR-100 promoted neurite growth in RGC-5 cells. (A)** RGC-5 cells were treated with miR-NC or miR100-inhibitor for 14 days, followed by immunostaining with an antibody against Thy-1 (green). DAPI (blue) staining was also performed. **(B)** Neurite lengths were measured using ImageJ software and compared between miR100-inhibitor treated RGC-5 cells and miR-NC treated RGC-5 cells (*, *P* <0.05).

Interestingly, once the culture period was extended to 14 days after lentiviral transfection, we discovered that the morphology of RGC-5 cells was dramatically modified by miR-100 down-regulation. The RGC-5 cells with down-regulated miR-100 extended the peripherals to be long and neurite-like processes, whereas the cells without miR-100 downregulation kept original morphology of progenitor cell with short peripheral processes (Figure 4A). Quantitative measurement with ImagJ software (http://imagej.nih.gov/ij/) confirmed the effect of down-regulating miR-100 on promoting neurite length in RGC-5 cells (Figure 4B, *: *P* <0.05).

### Downregulation of miR-100 phosphorylated AKT/ERK/TrkB pathways in RGC-5 cells

**Figure 5:**
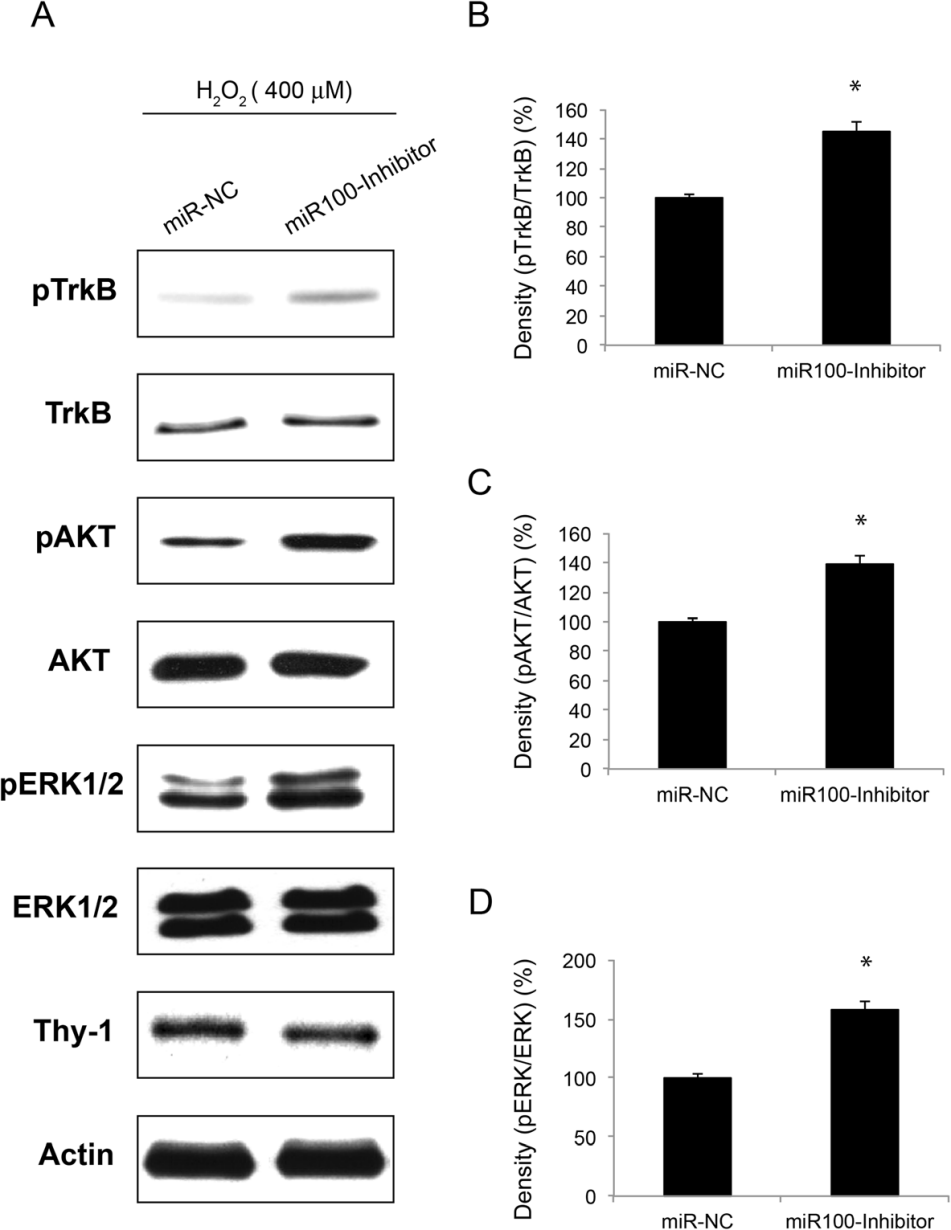
**Effects of down-regulating miR-100 on AKT/ERK/TrkB pathways in RGC-5 cells.** RGC-5 cells were pre-treated with miR100-inhibitor, or miR-NC for 24 hours, followed by H_2_O_2_ (400 μM) treatment for another 24 hours. **(A)** Western blotting analysis was performed to examine the protein expressions of Thy-1, ERK1/2, phspho-ERK1/2 (pERK1/2), AKT, phospho-AKT (pAKT), TrkB and phosphorylated-TrkB (pTrkB), in miR100-inhibitor pre-treated and miR-NC pre-treated RGC-5 cells. Semi-quantitative examination was performed to compare the phosphorylation levels of TrkB **(B)**, AKT **(C)** and ERK **(D)**. (*, *P* <0.05)

We then investigated the intracellular signaling pathways that might be involved in the protection of miR-100 down-regulation on RGC-5 apoptosis. In order to do that, RGC-5 cells were pre-treated with either miR100-inhibitor or miR-NC for 24 hours, followed by H_2_O_2_ (400 μM) treatment for another 24 hours. Western blotting analysis was used to measure the protein expression levels of Thy-1, ERK1/2, AKT and TrkB, as well as phosphorylation of ERK1/2, AKT and TrkB (Figure 5A). Our results demonstrated that down-regulating miR-100 did not alter the expression of Thy-1. However, miR-100 downregulation in RGC-5 cells significantly increased the phosphorylation in ERK1/2, AKT and TrkB, while keeping the total protein levels of ERK1/2, AKT and TrkB unchanged (Figure 5B-D, *: *P* <0.05).

### IGF1R was involved in miR-100 regulation in RGC-5

Finally, we intended to explore the possible molecular target of miR-100 modulation in RGC-5 cells. Through online bioinformatic screening (Target Scan, http://www.targetscan.org/), we noticed that, insulin-like growth factor 1 receptor (IGF1R) was a likely target of rat miR-100 (Figure 1A). We thus applied a luciferase reporter assay to verify that miR-100 was directly binding IGF1R in RGC-5 cells (Figure 1B).

We then speculated IGF1R might be directly involved in the modulation of miR-100 on RGC-5 apoptosis. In order to examine that, we constructed siRNA to specifically knock down IGF1R gene in RGC-5 cells (IGF1R-siRNA). After applying 100 nM IGF1R-siRNA or its non-specific control siRNA (NC-siRNA) in RGC-5 cells for 48 hours, we examined the knocking down efficiency by western blotting analysis. Our results showed that IGF1R protein was significantly reduced by IGF1R-siRNA (Figure 1C). The results also showed that down-regulation of IGF1R induced phosphorylation of AKT, whereas the total protein of AKT was unchanged (Figure 1C).

Finally, we examined the effect of knocking down IGF1R on miR-100 regulation and RGC-5 apoptosis. We pre-treated RGC-5 cells with either IGF1R-siRNA (100 nM) or NC-siRNA (100 nM) for 24 hours, followed by H_2_O_2_ (400 μM) treatment for another 24 hours. The results showed that the mRNA expression level of miR-100, presumably up-regulated upon apoptosis, was significantly down-regulated by knocking down IGF1R in RGC-5 cells (Figure 1D). Moreover, the percentage of apoptotic RGC-5 was markedly reduced by knocking down IGF1R, from 78% to 33% (Figure 1E).

## Discussion

Oxidative stress has been suggested to be a major mechanism causing RGC degeneration [1], and the application of H_2_O_2_ is known to induce apoptosis among cultured RGCs. In the present study, we utilized an *in vitro* culture system to induce RGC-5 apoptosis through H_2_O_2_ and found that miR-100 was up-regulated by H_2_O_2_ in a dose-dependent manner. It has been commonly shown in the literature that miR-100 is an active factor in cancer regulation, modulating carcinoma development, metastasis or apoptosis [25,27]. In retina, though miR-100 is expressed, little is known of its molecular mechanism [28]. In the present study, not only did we show an active change in expression profile of miR-100 upon H_2_O_2_-induced apoptosis, but we also demonstrated that down-regulation of miR-100 had a protective effect on RGC-5 apoptosis. Thus, these results highlighted a first ever report on functional role of miR-100 in retina.

Also in the present study we showed that, during H_2_O_2_-induced apoptosis, down-regulating miR-100 activated TrkB signaling pathways and its downstream Akt/ERK pathways, through phosphorylation. Thus, it is likely that miR-100 downregulation may serve a role of TrkB agonist to exert a protective effect on H_2_O_2_-induced apoptosis in RGC-5 cells. This hypothesis was further supported by our results showing miR-100 down-regulation promoted neurite growth, possibly induced neural differentiation in RGC-5 cells, as TrkB activation is known to induce maturation in retina [29]. However, to elucidate the exact molecular interaction between miR-100 and TrkB/Akt/ERK pathways in protecting retinal ganglion apoptosis, future experiments of blocking TrkB or its downstream pathways upon the inhibition of miR-100 would provide solid evidence on direct targeting of miR-100 on anti-apoptotic pathways in retinal ganglion cells.

Through online bioinformatics search, as well as our luciferase assay and functional experiments (Figure 1), we further revealed that IGF1R was very likely to be directly involved in the modulation of miR-100 on H_2_O_2_-induced apoptosis in RGC-5 cells. Furthermore, our results demonstrated that siRNA-mediated IGF1R knockdown activated Akt pathway and rescued H_2_O_2_-induced apoptosis. This is consistent with previous study showing that IGF1R mutant mice had increased lifespan and significant resistance to oxidative stress in retinal ganglion cells [8]. Interestingly, our experiment also showed that knocking down IGF1R was able to down-regulate miR-100. This result suggests that, instead of being downstream target of miR-100, IGF1R might be mutually interacted with miR-100 in regulating RGC apoptosis, acting through miR-100 itself or its upstream pathways. Along with our early results demonstrating the functional association between miR-100 and Akt/ERK/TrkB pathways, a much more complex molecular network might be involved in the regulation of miR-100 in retina.

## Conclusions

Overall, our study identified a novel regulator, miR-100 in modulating apoptosis in retinal ganglion cells. The method of down-regulating miR-100 might help to further our understanding on the mechanisms of degeneration and regeneration in retina tissues.
